# Lipidomic analyses reveal distinctive variations in homeoviscous adaptation among clinical strains of *Acinetobacter baumannii*, providing insights from an environmental adaptation perspective

**DOI:** 10.1128/spectrum.00757-24

**Published:** 2024-09-10

**Authors:** Clara Dessenne, Benoît Ménart, Sébastien Acket, Gisèle Dewulf, Yann Guerardel, Olivier Vidal, Yannick Rossez

**Affiliations:** 1 Université Lille, CNRS, UMR 8576-UGSF-Unité de Glycobiologie Structurale et Fonctionnelle, Lille, France; 2 Centre Hospitalier de valenciennes, Laboratoire de Biologie Hygiène—service de Microbiologie, Valenciennes, France; 3 Université de technologie de Compiègne, UPJV, UMR CNRS 7025, Enzyme and Cell Engineering, Centre de recherche Royallieu, Compiègne Cedex, Compiègne, France; 4 Institute for Glyco-core Research (iGCORE), Gifu University, Gifu, Japan; Emory University School of Medicine, Atlanta, Georiga, USA

**Keywords:** *Acinetobacter baumannii*, phospholipids, motility, temperature adaptation, biofilms

## Abstract

*Acinetobacter baumannii* is known for its antibiotic resistance and is increasingly found outside of healthcare settings. To survive colder temperatures, bacteria, including *A. baumannii*, adapt by modifying glycerophospholipids (GPL) to maintain membrane flexibility. This study examines the lipid composition of six clinical *A. baumannii* strains, including the virulent AB5075, at two temperatures. At 18°C, five strains consistently show an increase in palmitoleic acid (C16:1), while ABVal2 uniquely shows an increase in oleic acid (C18:1). LC-HRMS2 analysis identifies shifts in GPL and glycerolipid composition between 18°C and 37°C, highlighting variations in phosphatidylethanolamine (PE) and phosphatidylglycerol (PG) lipids. ABVal2 shows increased PE with C18:1 and C16:1 at 18°C, but no change in PG, in contrast to other strains that show increased PE and PG with C16:1. Notably, although *A. baumannii* typically lacks FabA, a key enzyme for unsaturated fatty acid synthesis, this enzyme was found in both ABVal2 and ABVal3. In addition, ABVal2 contains five candidate desaturases that may contribute to its lipid profile. The study also reveals variations in strain motility and biofilm formation over temperature. These findings enhance our understanding *of A. baumann*ii’s physiological adaptations, survival strategies and ecological fitness in different environments.

**IMPORTANCE:**

*Acinetobacter baumannii*, a bacterium known for its resistance to antibiotics, is a concern in healthcare settings. This study focused on understanding how this bacterium adapts to different temperatures and how its lipid composition changes. Lipids are the building blocks of cell membranes. By studying these changes, scientists can gain insights into how the bacterium survives and behaves in various environments. This understanding improves our understanding of its global dissemination capabilities. The results of the study contribute to our broader understanding of how *Acinetobacter baumannii* works, which is important for developing strategies to combat its impact on patient health.

## INTRODUCTION

*Acinetobacter* spp., a versatile and resilient group of bacteria, has gained significant attention in recent years due to mainly *Acinetobacter baumannii* which is commonly found in healthcare settings and long-term care facilities (1). This bacterium, a member of the *Acinetobacter calcoaceticus-baumannii* (Acb) complex, is implicated in human infections, alongside other species such as *A. lwoffi*, *A. junii*, *A. nosocomialis*, and *A. pittii*. Additionally, *A. calcoaceticus* is regarded as an environmental species (2). One of the biggest concerns with *A. baumannii* is its ability to develop resistance to multiple antibiotics, which can make infections difficult to treat (3). Although *Acinetobacter* spp. are renowned for their widespread presence and exceptional adaptability, the case appears to differ when it comes to *A. baumannii*. In contrast to other *Acinetobacter* species commonly found in soil or water samples, *A. baumannii* was believed to be rarely isolated from such sources, unless it was inadvertently introduced through human waste (4, 5). However, over the past decade, research has shed light on the presence of *A. baumannii* in non-clinical environments, rekindling the mystery surrounding its relative lack of environmental adaptability when compared to other *Acinetobacter* species (6–9). Bacterial human pathogens, like *A. baumannii*, must undergo specific physiological adaptations to endure outside the human body, and these adaptations can exert a profound influence on their pathogenicity (10, 11). Among the known mechanisms, the viable but non-culturable (VBNC) state allows bacteria to survive prolonged periods of unfavorable conditions (12). Another example is homeoviscous adaptation, a common response to low temperatures that allow bacteria to survive extracorporeal conditions. This adaptation involves modifying the composition of glycerophospholipids (GPL) by altering their fatty acids, ultimately enhancing their flexibility (13). Therefore, the bacterial cell membrane plays a crucial role in providing a protective barrier against the external environment and importantly to antibiotic resistance in several bacterial species (14–16). GPL in *A. baumannii* are found through seven sub classes including phosphatidylethanolamine (PE), phosphatidylglycerol (PG), lysophosphatidylethanolamine (LPE), hemibismonoacylglycerophosphate (HBMP), cardiolipin (CL), monolysocardiolipin (MLCL), and phosphatidylcholine (PC) (17, 18). *A. baumannii* contains two glycerolipids (GL), namely, triacylglycerol (TG) and diacylglycerol (DG) (17). These GL serve not only as reserve compounds but also fulfill roles in metabolism, potentially acting as sources of fatty acids that can be readily mobilized (19).

Within a spectrum of survival mechanisms, bacteria possess the capacity to form biofilms or demonstrate motility as strategies for dispersion and adaptation within their environmental niches. Additionally, motility also plays a significant role in actively moving toward and colonizing specific sites within the host (20). Two distinct types of motility have been characterized in *Acinetobacter* spp.: twitching motility, which relies on the type IV pilus for movement (21), and surface-associated motility. The latter is influenced by various factors, including the PrpABCD pilus and 1,3 diaminopropane (22–25). In addition to motility, *A. baumannii*’s capability to form biofilms further contributes to its pathogenicity and persistence by making eradication more challenging (26, 27). Interestingly, the impact of temperature on the physiology and virulence of *A. baumannii* remains relatively understudied while these nosocomial bacteria rely on a dynamic interplay between their ability to colonize the human body and their capacity to spread within the hospital environment.

*A. baumannii* shows high genetic diversity. Reference strains do not represent the full range of clinical isolates, so strain selection is crucial. The use of contemporary isolates should be preferred over historical strains to ensure accurate research on virulence and drug resistance (28). In this article, five strains of *A. baumannii* isolated from patients in intensive care in France are compared with the highly virulent model strain, AB5075, isolated in 2008 from a combatant wound infection (29, 30). The primary objective of this research is to investigate how clinical strains of *A. baumannii* can adapt to both environmental temperature and human body temperature. To address this, our initial goal is to investigate changes in fatty acid content among these six strains when they are cultivated at both 37°C, which is a standard temperature for growing human pathogens, and 18°C, representing an environmental temperature typical in temperate countries, compatible with the occurrence of *A. baumannii* in livestock animals (7, 31). Notably, one of the six strains exhibited distinct behavior compared to the others, and subtle differences were observed among the remaining five strains. Subsequently, we endeavor to conduct a comprehensive analysis of the lipid composition of these strains by utilizing liquid chromatography-high-resolution tandem mass spectrometry (LC-HRMS2), with a particular focus on GPL and GL. Additionally, we examined the influence of temperature on motility, biofilm formation capabilities, doubling time, and membrane fluidity in these six distinct *A. baumannii* strains. Finally, using whole-genome sequencing, we identified the insertion of several genes from the bacterial type II fatty acid biosynthetic pathway only in ABVal2 and ABVal3 genomes. Essentially, this research seeks to unveil potential variations in homeoviscous adaptation among different *A. baumannii* strains and explore further into the influence of temperature on their physiology. By doing so, it aims to illuminate the ecological versatility and adaptive mechanisms of this organism.

## RESULTS

### Bacterial species-level identification, sequence types, and antibiotic resistance pattern

Five strains of *Acinetobacter* spp. were isolated from France and specifically selected to represent different antibiotic resistance profiles. Additionally, a reference strain, AB5075, isolated from a patient with osteomyelitis in the United States was included in this study (29). To identify the five isolated strains, matrix-assisted laser ionization time-of-flight mass spectrometry (MALDI-TOF MS) has been used. This method has been effectively utilized in clinical microbiology labs for rapid bacterial identification. However, this technique is unable to accurately identify species within the Acb complex (32). Although all strains were initially identified as *A. baumannii*, we conducted amplification and sequencing of the 16S-23S rRNA gene spacer region (33) to confirm the identity of the strains used in this study. Fragments of approximately 600 bp were observed, exhibiting a high degree of similarity among the six strains, including AB5075 (Fig. S1). In pursuit of a more comprehensive understanding of the phenotypic diversity among the various strains and the potential for distinct antibiotic resistance profiles, we conducted antibiograms using the antibiotics commonly employed in routine clinical practice in France. As indicated in Table 1, the strain AB5075 exhibits resistance to nearly all antibiotics, with the exception of minocycline, as previously described (29). ABVal3 is resistant to all antibiotics tested, whereas ABVal2 requires higher concentrations of Ticarcillin, Trimethoprim-sulfamethoxazole, and three β-lactams (clavulanic acid with tricarcillin, cefepime, and imipenem) to exhibit susceptibilities. ABVal1 is susceptible to Trimethoprim–sulfamethoxazole only while ABVal4 is resistant to Aztreonam, Fosfomycin, and at low concentration to Ciprofloxacin. We further analyzed the five strains for their respective genetic backgrounds to generate *de novo* assembled genomes using sequencing data from Oxford Nanopore Technologies. We next analyzed the alleles to determine the sequence types (ST) according to the Oxford (Oxf) and Pasteur (Pas) schemes. For the Oxf scheme, the results for ABVal1, 2, 3, 4, and 5 were ST1418, 1806/208, 1816/195, 1604/231, 1037, and 1677/945, respectively. Using the Pas scheme, the corresponding results were ST164, 2, 2, 1, and no data were obtained for ABVal5. For AB5075, the sequence types were STOxf1677/945 and STPas1. For more information, see Table S1.

**TABLE 1:** Antibiotic resistance exhibited by the strains used in this study^*a*^

Antibiotic group	Antibiotics	ABVal1	ABVal2	ABVal3	ABVal4	ABVal5	AB5075
Carboxypenicillin	Ticarcillin	R	S (at high concentration)	R	S	R	R
Carboxypenicillin—β-lactam	Ticarcillin—clavulanic acid	R	S (at high concentration)	R	S	S	R
β-Lactam	Piperacillin	R	R	R	S	R	R
β-Lactam—β-lactamase inhibitor	Piperacillin—Tazobactam	R	R	R	S	R	R
β-Lactam	Aztreonam	R	R	R	R	R	R
β-Lactam	Ceftazidime	R	R	R	S	R	R
β-Lactam	Cefepime	R	S (at high concentration)	R	S	R	R
β-Lactam	Imipenem	R	S (at high concentration)	R	S	S	R
Fluoroquinolone	Levofloxacin	R	R	R	S	R	R
Fluoroquinolon	Ciprofloxacin	R	R	R	S (at high concentration)	R	R
Aminoglycoside	Gentamicin	R	R	R	S	R	R
Aminoglycoside	Tobramycin	R	R	R	S	R	R
Aminoglycoside	Amikacin	R	R	R	S	R	R
Tetracycline	Minocycline	R	R	R	S	S	S
Phosphonic	Fosfomycin	R	R	R	R	R	R
Antifolate—Sulfonamide	Trimethoprim—sulfamethoxazole	S	S (at high concentration)	R	S	R	R

^*a*^ R indicates resistance to the corresponding antibiotic and S stands for susceptible.

### Fatty acids content at different temperatures

**Fig 1:**
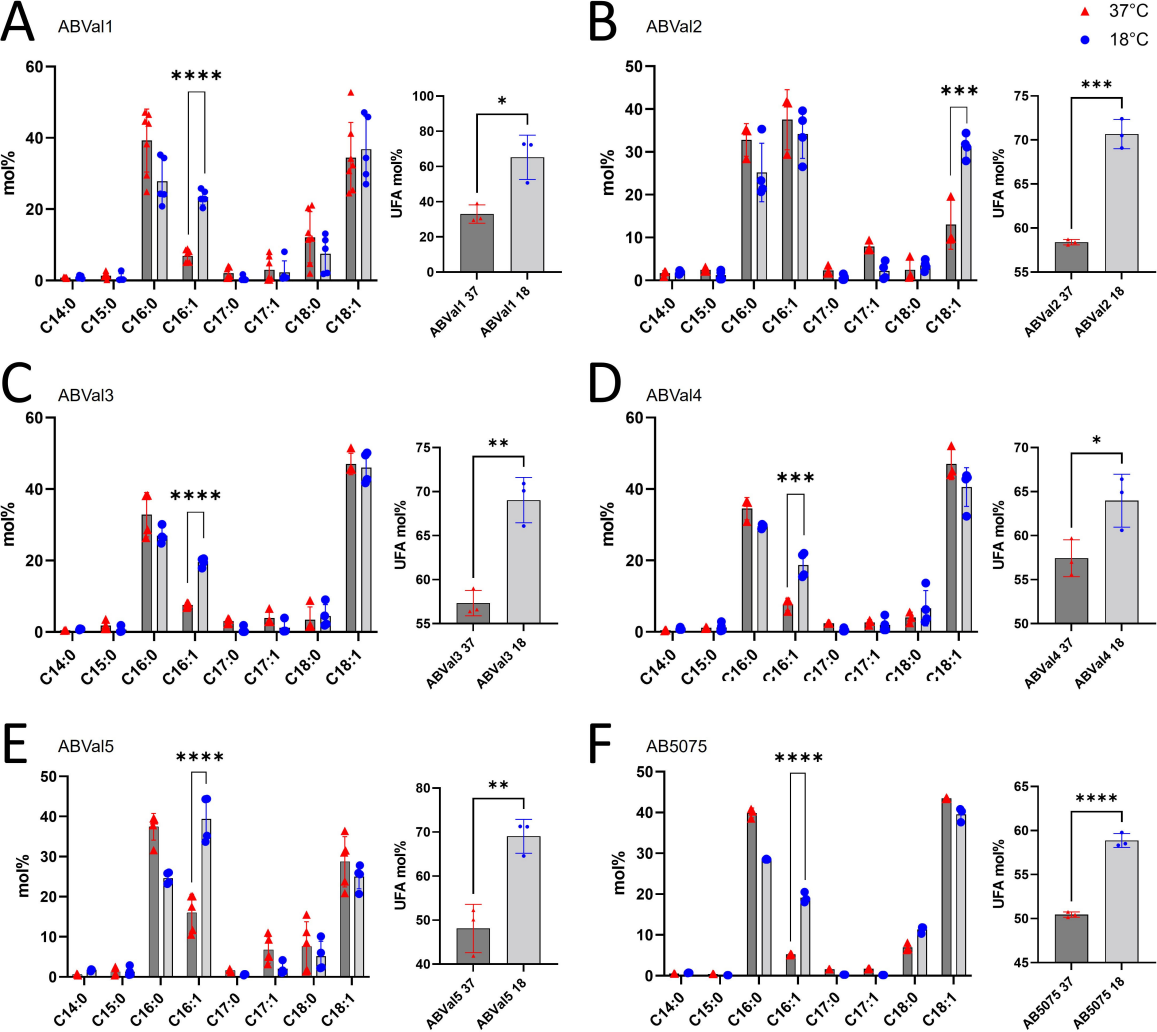
Fatty acid methyl ester analysis of *A. baumannii* clinical strains by GC-FID cultivated at 37°C or 18°C. The results correspond to *n* = 3 biologically independent samples. (**A**) ABVal1, (**B**) ABVal2, (**C**) ABVal3, (**D**) ABVal4, (**E**) ABVal5, (**F**) AB5075. %UFA was calculated for each strain in mol%. Statistical significances were determined by a two-tailed student’s *t* test (****P ≤ 0.0001; ***P ≤ 0.001).

To estimate the temperature adaptation of the six strains, the fatty acid content was determined using gas chromatography with a flame ionization detector (GC-FID) after culturing them in LB at 37°C and 18°C (Fig. 1). At 37°C, the major fatty acids detected were palmitic acid (C16:0) and oleic acid (C18:1), comprising approximately 35%–40% and 30%–40% of the total fatty acids, respectively. ABVal2, however, exhibited approximately 30% and 10% of these two fatty acids. Interestingly, ABVal2 also displayed a higher percentage of palmitoleic acid (C16:1) compared to other strains, with levels around 40% (Fig. 1B). C16:1 content varied between 5% and 15% at 37°C in the other strains, while the remaining fatty acids were below 5%, consistent with previous observations (17). At 18°C, a significant increase in C16:1 content was observed in strains ABVal1, ABVal3, ABVal4 and ABVal5, and AB5075 compared to 37°C (Figure A, C, E, and F, respectively). To further elucidate the distinct characteristics observed at 37°C and 18°C, especially in the case of ABVal2, we expanded our investigation to include the GPL and GL composition beyond mere fatty acid content. This exploration involved the application of LC-HRMS2 analysis.

### LC-HRMS^2^ analyses and temperature adaptations

**Fig 2:**
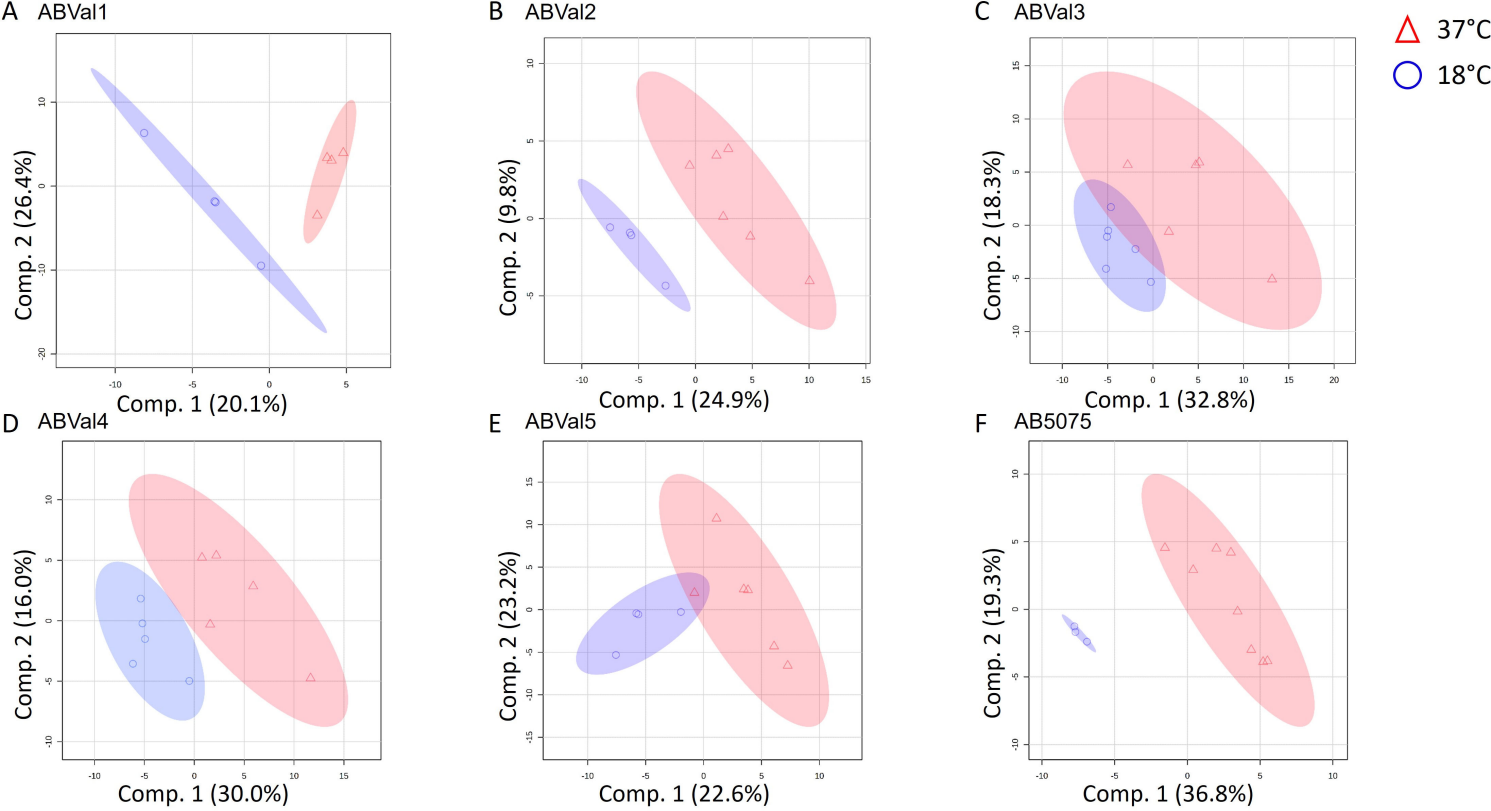
Partial least squares-discriminant analysis (PLS-DA) scores plot showing variances in lipid species between 37°C and 18°C for the strains (**A**) ABVal1, (**B**) ABVal2, (**C**) ABVal3, (**D**) ABVal4, (**E**) ABVal5, (**F**) AB5075. The results correspond to at least three biologically independent samples. The analysis was performed using MetaboAnalyst V5.0 (https://www.metaboanalyst.ca/, accessed in May 2023).

**Fig 3:**
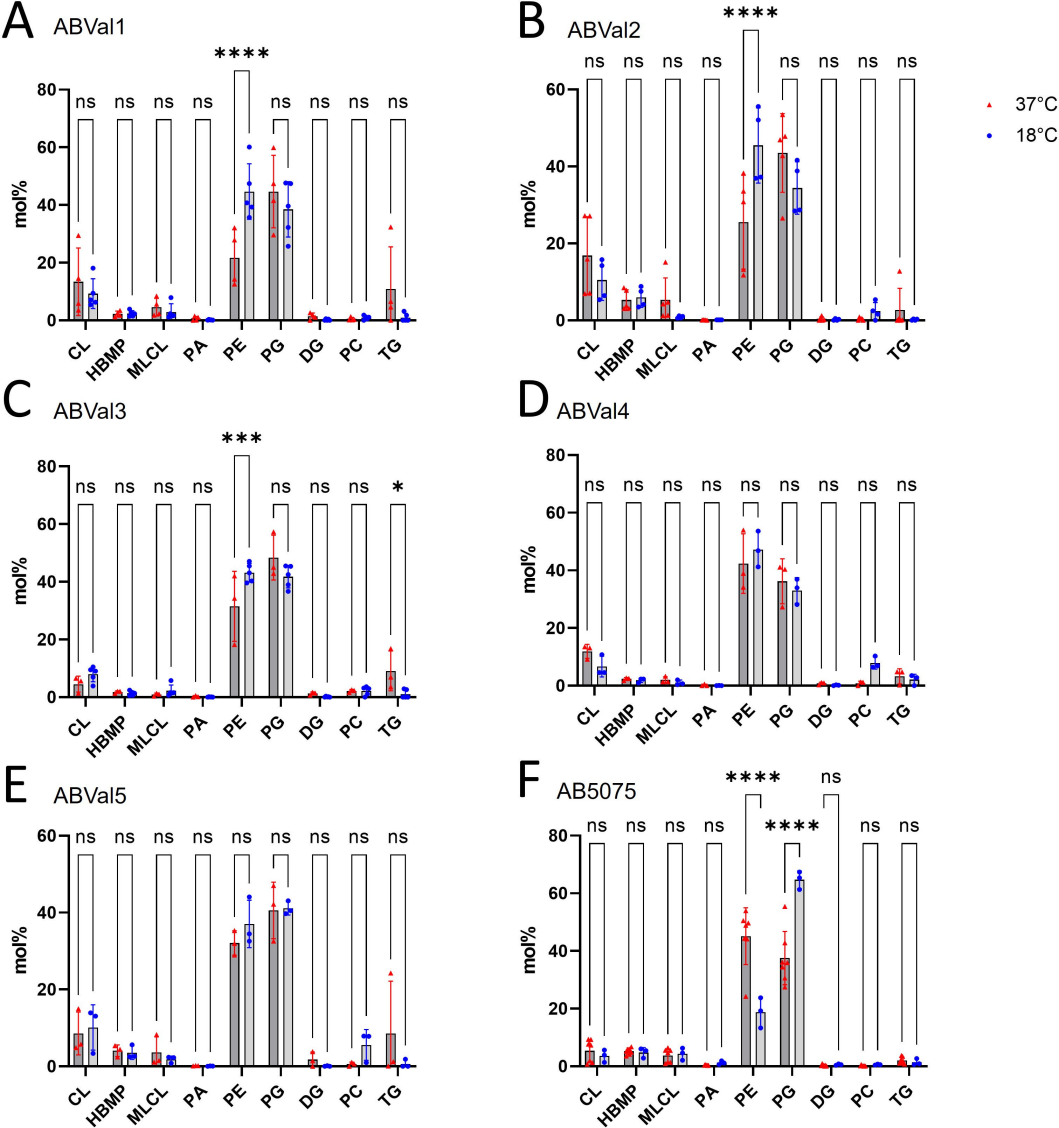
Glycerophospholipids and glycerolipids percentages of *A. baumannii* clinical strains cultivated at 37°C or 18°C based on LC-HRMS^2^ data with (**A**) ABVal1, (**B**) ABVal2, (**C**) ABVal3, (**D**) ABVal4, (**E**) ABVal5, (**F**) AB5075. The results correspond to at least three biologically independent samples. Statistical significances were determined by *post-hoc* Tukey test after two-way analysis of variance (****P ≤ 0.0001; ***P ≤ 0.001; ns, *P* > 0.05). CL, cardiolipin; HBMP, hemibismonoacylglycerophosphate; MLCL, monolysocardiolipin; PA, phosphatidic acid; PE, phosphatidylethanolamine; PG, phosphatidylglycerol; DG, diacylglycerol; PC, phosphatidylcholine; TG, triacylglycerol.

Partial least squares-discriminant analysis (PLS-DA) was utilized to analyze the lipidome data and determine if there was any discernible separation among each strain when exposed to 37°C and 18°C (Fig. 2). While there were variations in the GPL and GL compositions between the two temperatures for all strains (Fig. 3), ABVal1, ABVal2, and AB5075 displayed more pronounced differences (Fig. 2A, B and F, respectively). Subsequently, to obtain a holistic understanding of each subclass, their respective proportions were calculated (Fig. 3). Across all strains, the prevailing lipids were predominantly PE and PG. Notably, there was a substantial rise in PE levels at 18°C for ABVal1, ABVal2, and ABVal3 (Fig. 3A through C, respectively). Conversely, in the case of AB5075, a reduction in PE content was discernible at 18°C, accompanied by a concurrent elevation in PG levels (Fig. 3F).

**Fig 4:**
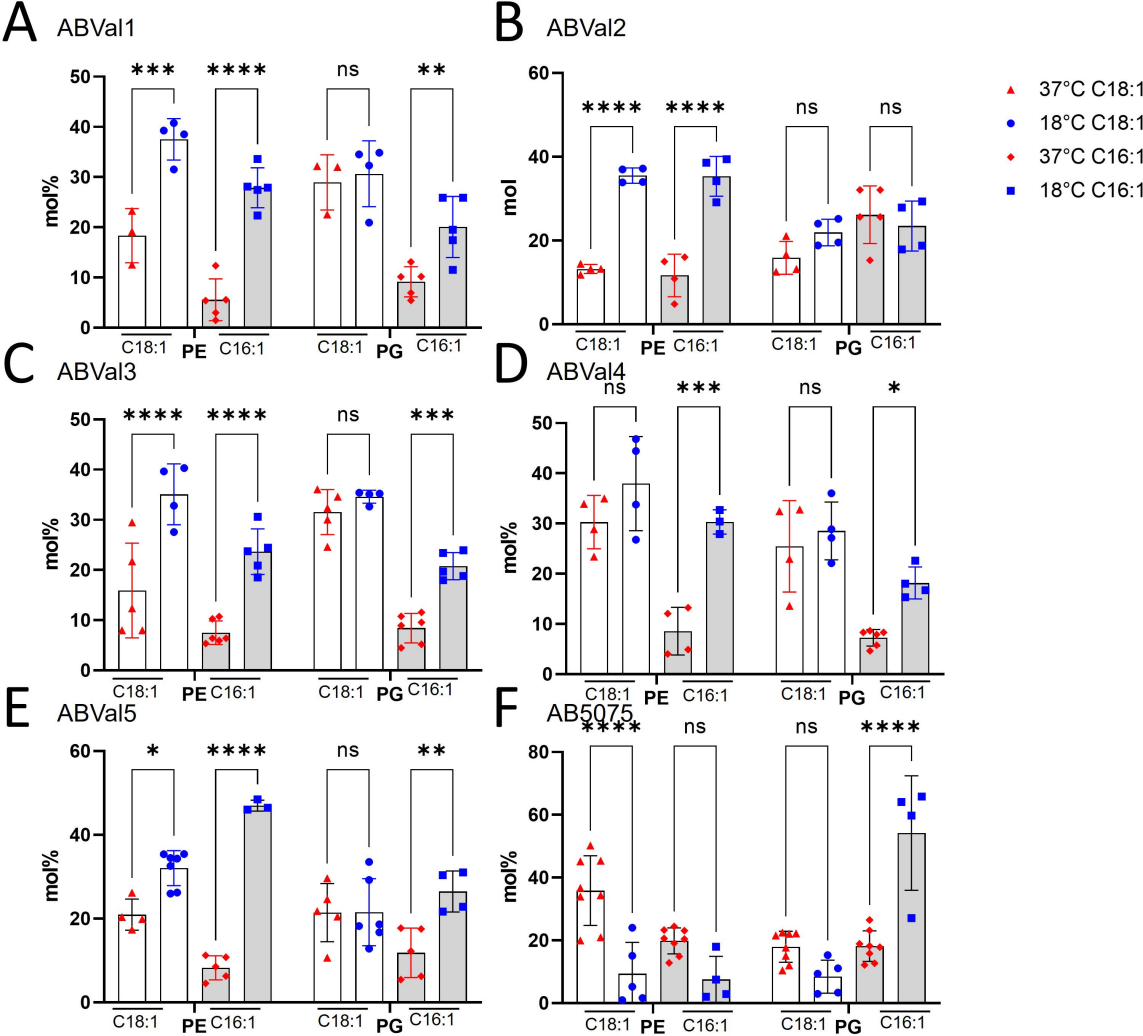
PE and PG containing at least one C16:1 or C18:1 percentages of *A. baumannii* clinical strains cultivated at 37°C or 18°C based on LC-HRMS^2^ data with (**A**) ABVal1, (**B**) ABVal2, (**C**) ABVal3, (**D**) ABVal4, (**E**) ABVal5, (**F**) AB5075. The results correspond to at least three biologically independent samples. Statistical significances were determined by *post-hoc* Tukey test after two-way analysis of variance (****P ≤ 0.0001; ***P ≤ 0.001; **P ≤ 0.01; *P ≤ 0.05; ns, *P* > 0.05). PE, phosphatidylethanolamine, PG, phosphatidylglycerol.

Given the prevailing abundance of PE and PG among the extracted lipids, the proportions of PE and PG molecules each incorporating one or two C18:1 and C16:1 fatty acids, which experience upregulation at 18°C, were computed and subsequently juxtaposed (Fig. 4). In the case of ABVal1, 2, 3, and 5, an elevated occurrence of PE containing C18:1 was discernible at 18°C, while no significant differences were apparent for PG of all strains. Conversely, for AB5075, the quantity of PE containing C18:1 exhibited a reduction (white bars in Fig. 4F). The identical observation was repeated for PE containing C16:1 including ABVal4 (represented by the gray bars in Fig. 4A through F, respectively). However, for PG containing C16:1, an elevation was noticed in ABVal1, 3, 4, 5, and AB5075. The more pronounced increase in PG C16:1 at 18°C for AB5075 was particularly noteworthy (as indicated by the gray bars in Fig. 4F). For ABVal2, no significant fluctuation in either PG containing C18:1 or C16:1 was discernible between the two temperatures (Fig. 4B).

### Bacterial responses to 18°C and 37°C: bacterial motilities, biofilm, growth, and membrane fluidity

**Fig 5:**
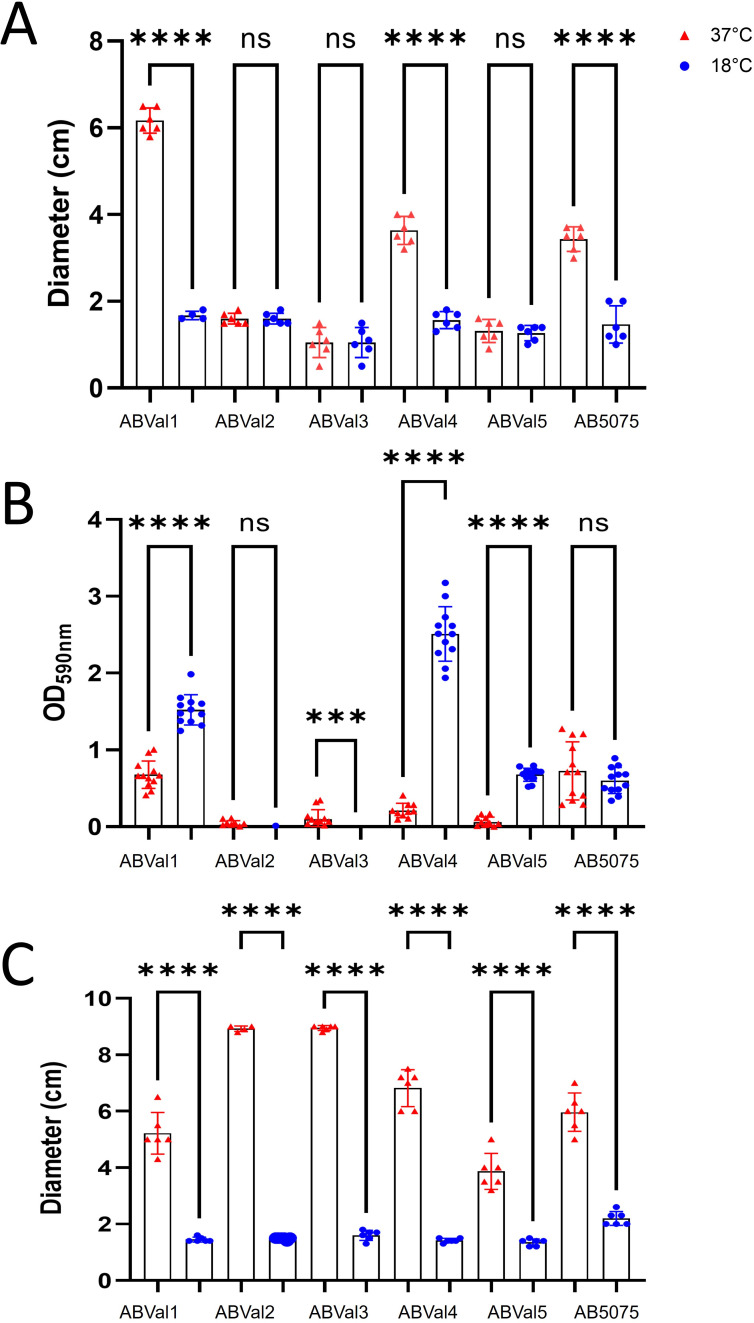
(**A**) Twitching motility at 37°C and 18°C after 48 hours of inoculation. (**B**) Biofilm formation at 37°C and 18°C. The graph represents the level of biofilm formation (absorbance at 590 nm), which was investigated in a 96-well microtiter tray using a crystal violet stain method, with data collected from either 10 or 12 wells. (**C**) Surface-associated motility at 37°C and 18°C after 48 hours of inoculation. The results are based on three biologically independent samples unless otherwise specified in the Materials and Methods section. Statistical significances were determined by a two-tailed student’s *t* test (****P ≤ 0.0001; ***P ≤ 0.001; ns, *P* > 0.05).

**Fig 6:**
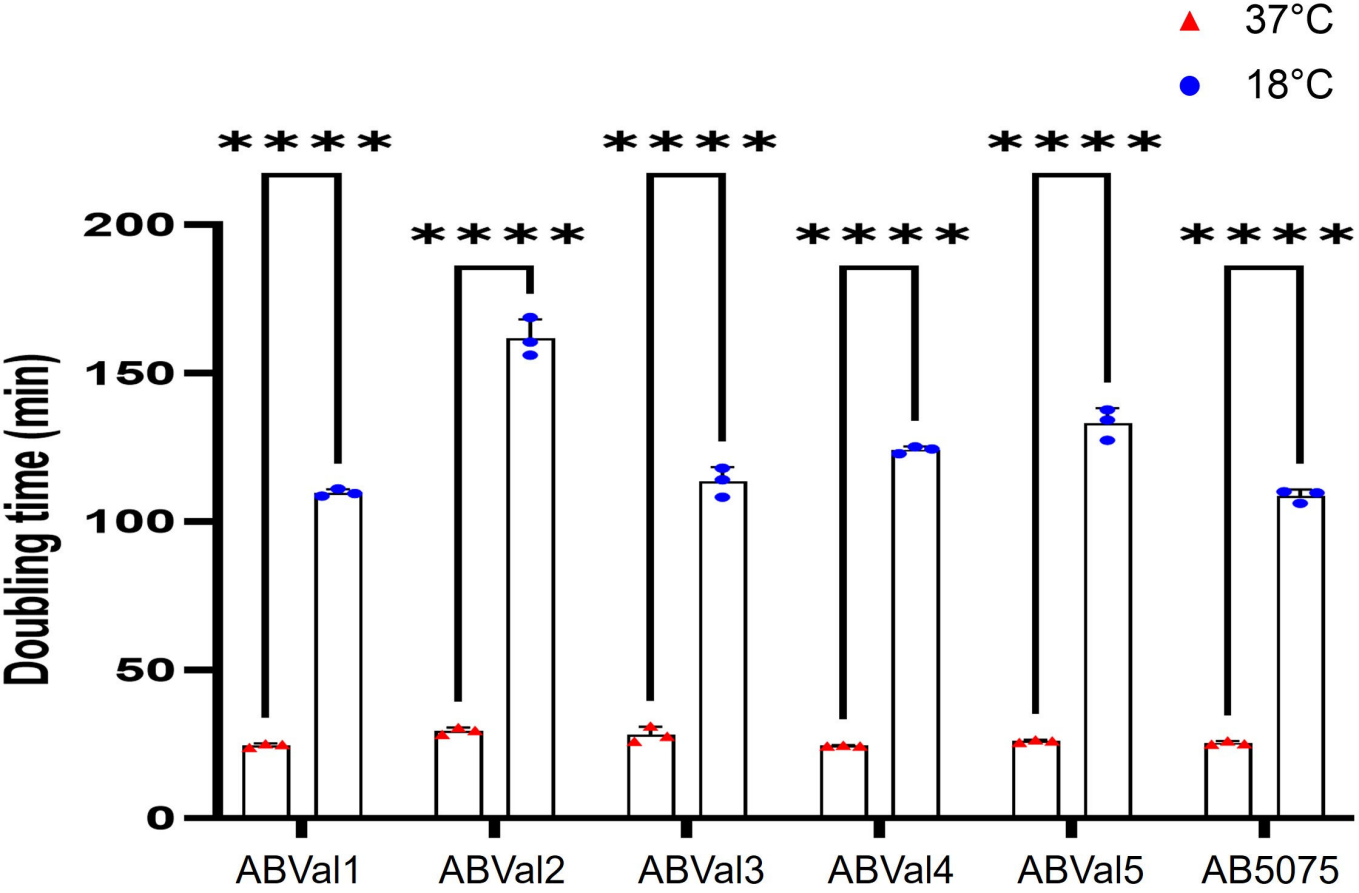
Doubling time of each strain at 37°C (red) and at 18°C (blue). The results are based on three biologically independent samples. Statistical significances were determined by a two-tailed student’s *t* test (****P ≤ 0.0001; **P ≤ 0.01; ns, *P* > 0.05).

To comprehensively assess bacterial behavior at both 37°C and 18°C, we evaluated the twitching motility of all six strains (Fig. 5A). Among the strains, ABVal2, ABVal3, and ABVal5 showed minimal motility at either temperature, whereas ABVal1, ABVal4, and AB5075 exhibited significant twitching motility at 37°C. Of these, ABVal1 displayed particularly efficient movement, covering a greater distance than the others at 37°C. Notably, no distinct twitching motility was observed at 18°C. To microscopically assess potential bacterial movement during twitching motility and discern movement during bacterial growth, AB5075 was observed under a microscope at both temperatures (Supplemental movie, Video S1). No movement was observed at 18°C, whereas the bacteria exhibited motility at 37°C (Supplemental movie, Video S2). Given that Type IV pili have been proposed to play a role in biofilm formation by facilitating initial bacterial attachment, a concept demonstrated in various bacterial species, including *A. baumannii* (21, 34, 35), we proceeded to evaluate each strain’s biofilm-forming capacity at both temperatures (Fig. 5B). ABVal2 and ABVal3 showed negligible biofilm formation at 18°C and modest biofilm production at 37°C. Interestingly, AB5075 exhibited consistent medium-level biofilm production that remained unaffected by the temperature variations. ABVal5 displayed medium biofilm production, but exclusively at 18°C, while its biofilm formation weakened significantly at 37°C. ABVal1 and ABVal4 emerged as robust biofilm producers at 18°C; however, their biofilm production reduced to a medium level at 37°C. As described before, there appeared to be an inverse correlation between surface-associated motility and the capacity to form biofilms (36). At 18°C, surface-associated motility was nearly nonexistent in all strains, whereas at 37°C, this motility was observable across all strains (Fig. 5C). Notably, ABVal2 and ABVal3 demonstrated the highest motility among the strains. For a comprehensive comparison of the six studied strains, all were cultured at both 18°C and 37°C (Fig. 6). At 37°C, ABVal2 exhibited a significantly slower doubling time of 29 minutes compared to AB5075, which had a doubling time of 25 minutes. At 18°C, a distinct difference was particularly evident, with ABVal2 showing a doubling time of 2 hours and 42 minutes, while AB5075 exhibited a faster doubling time of 1 hour and 49 minutes. To a lesser extent, ABVal4 also displayed a significant delay compared to AB5075, with a doubling time of 2 hours and 4 minutes. To correlate the extended doubling time of ABVal2 with membrane functionality, generalized polarization (GP) measurements were performed using laurdan (6-dodecanoyl-2-dimethylaminonaphthalene). Laurdan is a fluorescent probe that integrates into the membrane bilayer and exhibits an emission wavelength shift due to the presence of water molecules in the membrane (37). A high GP generally indicates low fluidity, and this trend was consistent across all strains, with a higher GP observed at 18°C (Fig. S2A). Membrane permeability was also evaluated among the strains and showed no significant differences. All strains showed increased dye uptake at lower temperatures (Fig. S2B).

### Genomic differences in genes associated with fatty acid desaturation

**Fig 7:**
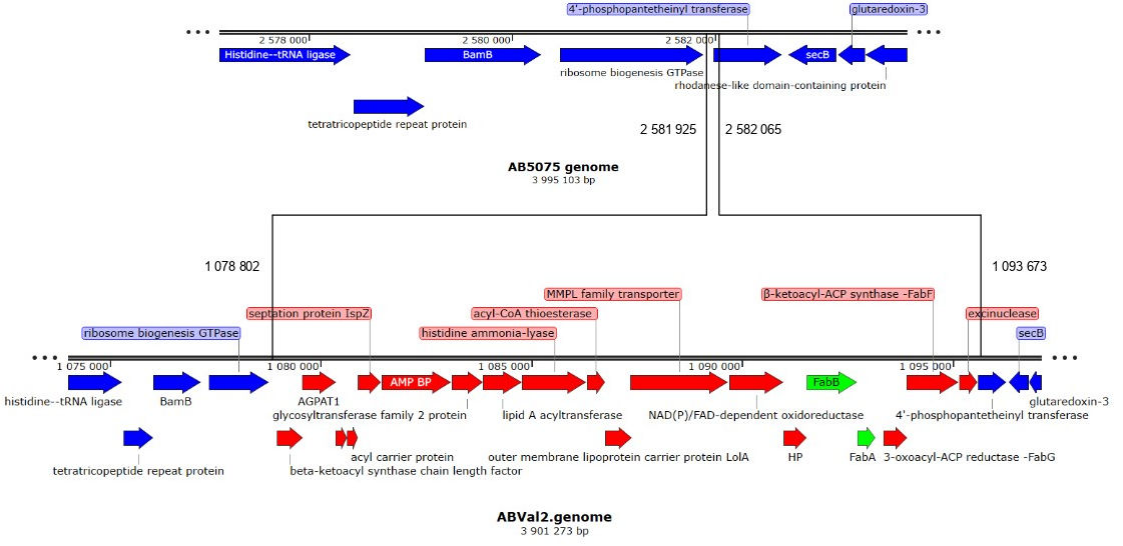
Genomic region of ABVal2 spanning nucleotides 1,078,802 to 1,093,673. Genes and annotations present in the insertion but absent from the AB5075 genome are highlighted in red and green.

To understand why ABVal2 exhibited a substantial increase in oleic acid content at 18°C compared to 37°C, we analyzed the genes involved in fatty acid desaturation, including fatty acid desaturases and genes from type II fatty acid synthesis (FASII), using genomic data from the five strains and data available on NCBI for AB5075. In *A. baumannii*, the desaturases DesA and DesB have been previously identified by others and are characterized for desaturating C16:0 for DesA (38). No differences in their sequence were observed for the two desaturases described among the strains (data not shown). However, three other candidates are homologous to *desA* and *desB* from *Pseudomonas aeuginosa* in ABVal2 (39) with two other potential DesA and one DesB (Fig. S3). For all of them three conserved histidine-rich motifs [“HX3 (or X4)H,” “HX2 (or X3)HH,” and “H/QX2 (or X3)HH”] are identified, and several transmembrane domains are present which are required for bacterial desaturase activities (40). The two *desB* genes are colocalized in an operon with an homolog of *desT*, a transcriptional regulator, and a putative oxidoreductase involved in the electron transport chain that supports the fatty acid desaturation reaction (39). In addition, *A. baumannii* is known from the literature to have an unusual FASII pathway because it lacks FabA. FabA catalyzes the dehydration of 3-hydroxydecanoyl-ACP to trans-2-decenoyl-ACP and can isomerize the double bond to cis-3-decenoyl-ACP, the precursor of unsaturated fatty acids (38, 41). Surprisingly, a genomic insertion containing FabB, FabG, and nearly 20 other genes was found in ABVal2 and ABVal3 (Fig. 7).

## DISCUSSION

Like other bacterial species, *A. baumannii* maintains its protective barrier’s integrity through well-coordinated lipid homeostasis pathways, resulting in a diverse array of GPL and GL molecules (42). These major GPLs not only preserve membrane integrity but also aid in forming domains that facilitate protein translocation, guide cell division locations, and influence antibiotic penetration (17, 43–46). Understanding how different *A. baumannii* strains adjust their lipid composition in response to temperatures similar to those found outside the human body is, therefore, crucial. ABVal4 exhibited a remarkable ability for twitching motility, which was particularly pronounced at 37°C, while its optimal biofilm formation potential was at 18°C. In this study, our aim was to compare the physiological adaptations of six clinically isolated strains in terms of their lipid contents, each characterized by unique antibiotic resistance profiles (Table 1), under conditions mimicking environmental and body temperatures. The range of documented lipid variations in *A. baumannii* upon exposure to lower temperatures is limited. As far as our knowledge extends, modifications in fatty acid content under cold conditions have only been described in lipooligosaccharides (47). The present work sheds light on the matter by uncovering a significant increase in palmitoleic acid (C16:1) content for ABVal1, ABVal3, ABVal4, ABVal5, and AB5075 at 18°C. Whereas, ABVal2 exhibited a notable elevation in oleic acid (C18:1) content at 18°C (Fig. 1). It is worth noting that the size of the fatty acids directly affects the fluidity of the plasma membrane. Longer fatty acid chains tend to decrease fluidity, while membranes containing unsaturated fatty acids are more loosely arranged and, therefore, more fluid (37, 48). ABVal2, with its elevated concentration of C18:1 at 18°C, may have reduced membrane fluidity compared to other strains, which could affect its adaptation to stress conditions at this temperature (Fig. 6) (49). However, the total unsaturated fatty acid content is between 60% and 80% for all strains at 18°C, which likely helps maintain an optimal level of fluidity as observed in the Laurdan experiment and membrane permeation studies (Fig. S2A and B). Curiously, an insertion of about 20 genes was identified in both ABVal2 and ABVal3, including *fabA* and *fabB*. These genes encode enzymes involved in fatty acid desaturation by introducing a double bond into a 10-carbon intermediate and are essential components of the FASII (Fig. 7). This intermediate is then elongated to form 16:1 and 18:1 fatty acids by FabF found in all strains (data not shown) (41, 50–52). A recent study revealed poor desaturase activity in *Pseudomonas putida*, likely due to a weakly active component involved in the electron transfer process (53). In ABVal2, the DesA DNA sequence is identical to that of AB5075. However, ABVal2 does not produce more C16:1 at 18°C, and the reason for this discrepancy may be due to FabA and FabB or related to an uncharacterized desaturase highlighted in the genome or to a poor electron transfer process of the desaturases.

Next, LC-HRMS2 analyses were performed to identify the specific lipids that could potentially accommodate the increased presence of unsaturated fatty acids at 18°C. First, a comprehensive assessment of total content was performed using PLS-DA to identify potential differences between different strains exposed to temperatures of 37°C and 18°C (Fig. 2). The outcomes highlighted notable shifts between the two temperatures for all strains. This observation aligns well with existing literature on bacterial adaptation facilitated by alterations in their lipid content (13, 54). In contrast to a previous study, LPE showed irregular detection across biological replicates, resulting in its omission from the analysis. Although phosphatidic acid (PA) was identified and included in this study, it represented only a fraction of the total lipid content (17) (Fig. 3).

Subsequently, we concentrated on the two principal unsaturated fatty acids, C18:1 and C16:1 (Fig. 1), which were upregulated at 18°C, specifically within the PE and PG lipids, known for their abundant presence (Fig. 4). This analytical approach enabled us to deduce which lipids subspecies were accountable for the distinct fatty acid profile observed in ABVal2 (Fig. 1). While PE containing C18:1 and C16:1 experienced upregulation at 18°C, no corresponding increase in PG with augmented C16:1 and C18:1 content was observed. This contrasted with all other strains, which exhibited elevated levels of PE and PG containing C16:1. However, AB5075 (Fig. 1F) showed no increase in PE containing C16:1 at 18°C. Notably, the increase observed is primarily attributed to PG at 18°C. Additionally, for AB5075, the diminishing levels of PE C18:1, PE C16:1, and PG C18:1 at 18°C indicate a discernible difference in lipid homeostasis compared to the other five strains under investigation. Hence, within the tested set of six strains, there are distinct behaviors observed: two strains exhibit dissimilar patterns, while the remaining four strains demonstrate notable similarity in terms of lipid synthesis under low temperatures. This phenomenon is a result of the significant gene repertoire diversity in *A. baumannii* and the notable influence of natural selection on protein evolution, driven by recombination and lateral gene transfer events within *A. baumannii* strains (55–58). Next, the study reveals variations in twitching motility and biofilm formation capacities among the six strains at different temperatures (Fig. 5). At lower temperatures, none of the strains exhibit motility, while three of them display enhanced biofilm-forming capabilities. ABVal1 showcases superior twitching motility at 37°C, with ABVal4 and AB5075 showing similar albeit lesser levels. Interestingly, these three strains have the shortest doubling times at 37°C (Fig. 6), suggesting that increased metabolic turnover increases energy availability in bacterial cells. This increased energy load could trigger ATP-dependent processes such as type IV pili dynamics and twitching motility since twitching motility requires ATP for the two ATPases involved in pilus extension and retraction (59). In terms of biofilm formation, ABVal2 and ABVal3 show minimal production, AB5075 consistently produces a moderate amount, ABVal5’s production varies with temperature, and ABVal1 and ABVal4 show moderate production at 37°C. The *Acb* complex is associated with higher infection rates during warmer periods, as documented in previous studies (60). This correlation supports findings that *A. baumannii* infection rates among hospitalized patients peak during warmer months (61). This phenomenon could be partly attributed to the temperature-sensitive regulation of motility, as evidenced by its decreased efficiency at lower temperatures. Similarly, studies of ATCC strain 17978, isolated in 1951 (62), have revealed an enhanced capability for biofilm formation at temperatures below 37°C, along with a reduction in twitching motility. In addition, *Ralstonia solanacearum*, which is known to induce wilt in several plant species, can induce symptoms in potatoes and tomatoes even at 18°C, but with reduced twitching motility (63). As noted above, there is an inverse relationship between surface-associated motility and biofilm formation, a point that is reiterated here (36, 64, 65). The motility and biofilm formation behaviors unique to each *A. baumannii* strain studied have been noted in previous studies of clinical strains (66–68). Reduced motility may hinder initial bacterial spread, while increased biofilm formation supports long-term persistence in various environments. Furthermore, as described for other appendages such as flagella, pili are components of the biofilm matrix. The type IV pili (T4P) can be part of this matrix and can form a larger part of it when the bacteria are less motile at lower temperatures. For adaptation to low temperatures, *A. baumannii* appears to benefit from increased production of C16:1, as observed in ABVal2. This strain, with its pronounced production of C18:1 at 18°C, has the slowest doubling time of the six strains (Fig. 6). ABVal2 and ABVal3 with the genomic insertion (Fig. 7), including FabA, belong to ST2 (from the Pasteur scheme, see Table S1), which is the predominant carbapenem-resistant *A. baumannii* lineage worldwide (69). Characterization of this phenotype in additional environmental and clinical strains could inform risk assessments for the spread of this species. These findings underscore the role of temperature in shaping lipid composition and fatty acid profiles and highlight strain-specific and temperature-dependent adaptations that may influence physiological properties, growth, and survival strategies.

## MATERIALS AND METHODS

### Strains and growth conditions

Clinical isolates were obtained from the microbiology department of the hospital of Valenciennes (France). All strains were isolated from the intensive care unit between 2014 and 2020. They were cultivated in lysogeny broth (LB)-Lennox (for 1 L 10 g of tryptone, 5 g of yeast extract, and 5 g of NaCl) at 18°C or 37°C at 180 rpm. Eighteen degree celcius was considered a relevant environmental temperature. The five strains were typed by *A. baumannii* Pasteur MLST scheme (70) and Oxford MLST scheme (71) using the BIGSdb software available at https://pubmlst.org/organisms/acinetobacter-baumannii/ (72).

### Whole-genome sequencing, assembly, and annotation

DNA from the five strains was isolated using a DNA purification kit Macherey Nagel, NucleoSpin Tissue, Mini kit for DNA from cells and tissue (reference: 740952.50). For sequencing, assembly, and annotation, Eurofins was utilized via the whole-genome sequencing service.

### Motility assays

All LB agar plates (Agar percentages are as follows: 1% concentration for twitching motility and 0.3% for surface-associated motility with Eiken chemical agar, Japan) were prepared freshly prior to being inoculated with 5 µL of a bacterial suspension in the exponential phase of growth (OD600: 0.3). For twitching motility, the inoculum was introduced by piercing the gel, while for surface-associated motility, the bacterial suspension was applied directly to the surface of the agar plate. For each temperature condition, the bacteria were initially cultured for a duration of 48 hours at either 37°C or 18°C accordingly. The diameter of the motility zone was recorded and analyzed, considering, while at the same time, the distance from the bottom of the medium to the plate for twitching motility, and at the surface of the plate for the surface-associated motility. To enhance result robustness, three independent measurements were executed for each strain, except for ABVal1 at 18°C and ABVal2 at 37°C. In these cases, two independent measurements were conducted for twitching and surface-associated motility, respectively. These measurements were carried out on two plates on the same day.

### Video of twitching motility

AB5075 was cultured in LB medium 2 hours before initiating the experiment to ensure that the bacteria were in the exponential growth phase. LB agar was poured into a tissue culture dish with a covered glass bottom (FluoroDish FD35-100), and inoculation was carried out following the procedure described for the motility assay. Microscopy was conducted using a Leica AF6000 LX inverted video microscope with differential interference contrast (DIC), capturing one image every 2 seconds for a duration of 2 minutes.

### Biofilm assays

Prior to initiating the experiments, the bacteria were cultured overnight at either 37°C or 18°C. Subsequently, 100 µL of LB medium containing bacteria in the exponential growth phase was aseptically transferred to individual wells of a 96-well plate. The methodology used here is based on previous work (73), with slight modifications. The adherent cells underwent a series of steps. Initially, they underwent three washes with phosphate-buffered saline (PBS) and were then left to air-dry in a cabinet for 2 hours. Following this, the cells were stained by incubating them with a solution of 0.1% crystal violet for 10 minutes. After staining, the wells were washed three times with PBS to eliminate any excess dye. For dye release from the biofilm, a solution of ethanol containing 10% acetone was employed. The released dye’s absorbance was measured at 590 nm using a plate reader. The presented biofilm data is an average taken from 10 or 12 wells across three independent biological samples. To assess the strains’ biofilm-forming ability, a modified version of a method derived from previous research (74) was employed. The average optical density at 590 nm (OD_590nm_) of 48 control wells incubated without bacteria at both 37°C and 18°C was determined as 0.28 (ODc). Using this control value, the bacteria’s biofilm-forming capability was classified as strong when the value was four times ODc, medium between two times ODc and four times ODc, low if below two times ODc, and absence of biofilm if the values were equal to or below ODc.

### Growth assays

Pre-cultures for each strain were prepared at either 37°C or 18°C, with the respective media initially incubated at the corresponding temperatures before inoculation. Sterile Erlenmeyer flasks (150 mL) containing 50 mL of LB medium were used, and each strain was added to achieve an initial Optical Density (OD) between 0.05 and 0.1. Measurements were taken in cuvettes using a spectrophotometer at a wavelength of 600 nm at intervals of 30 minutes to 1 hour and 30 minutes. The generated graphs displayed exponential growth phases, and the equations of the lines, represented in the form *y* = *ax* + *b*, where *y* = population and *x* = ime, were derived from these phases. Log (2) and Log (4) represent the doubling of the bacterial population. To compute the generation time, the formula utilized was: Generation Time = *x*2 – *x*1, where Generation Time = ((log (2)−*b*)/*a*) − ((log (4)−*b*)/*a*).

### Antimicrobial susceptibility testing

The disk diffusion method was performed according to norms of Clinical Laboratory Standards Institute (CSLI) (75). Antibiotics tested included Ticarcillin (75 µg), Ticarcillin —clavulanic acid 7.5:1 (85 µg), Piperacillin (100 µg), Piperacillin—Tazobactam 1:1 (110 µg), Aztreonam (30 µg), Ceftazidime (30 µg), Cefepime (30 µg), Imipenem (10 µg), Levofloxacin (5 µg), Ciprofloxacin (5 µg), Gentamicin (10 µg), Tobramycin (10 µg), Amikacin (30 µg), Minocycline (30 µg), Fosfomycin (200 µg), Trimethoprim—sulfamethoxazole 1:19 (25 µg)

### MALDI-TOF identification

After an incubation of 72 h at 37°C, single colonies were observed, and the bacterial species were identified by matrix-assisted laser desorption ionization-time-of-flight mass spectrometry (MALDI-TOF MS). Fresh colony material was spread on a MALDI target plate (MSP 96 target polished steel BC) (Bruker Daltonik GmbH, Germany) using a toothpick, mixed with 1 µL of a saturated α-cyano-4-hydroxy-cinnamic acid matrix solution in acetonitrile 50%–trifluoroacetic acid 2.5% and dried in air at ambient temperature. Mass spectra were acquired and analyzed on a microflex LT/SH mass spectrometer (Bruker Daltonik) using a Bruker’s MALDI Biotyper software reference database library of 3995 entries, version 3.1.2.0 and default parameter settings, as reported.

### PCR

Amplification of 16S rRNA and 23S rRNA was performed with the specific universal primers 1512F (5GTCGTAACAAGGTAGCCGTA3) and 6R (5GGGTTYCCCCRTTCRGAAAT3) as described previously (33). For DesA sequencing, the following primers were used DesA_forward: CACTCAAGGCCCCAATTAAC and DesA_reverse: TTCTAAACACTCACGGTGATG.

### Lipid extraction

Lipids were extracted using the Bligh and Dyer method with a mixture of methanol (MeOH), chloroform (CHCl3), and water (H_2_O) in a volumetric ratio of 2:1:0.8 as described previously (17). The extracted lipids were stored at −20°C until further analyses.

### Fatty acids analysis

The purified lipids were suspended in chloroform and a mixture of H2SO4 in methanol, butylated hydroxytoluene (BHT), and toluene was added and heated. Fatty acid methyl esters (FAME) were extracted using sodium chloride and heptane. The FAME composition was determined using gas chromatography, on a BPX70 column as described previously (17).

### LC-HRMS^2^, data processing, and annotation

The liquid chromatography used a Waters Aquity UPLC C18 column (100 × 2.4 mm, 1.7 µm) coupled to an Acquity UPLC CSH C18 VanGuard precolumn (5 × 2.1 mm; 1.7 µm) at 65°C. The mobile phase 60:40 (vol/vol) acetonitrile/water (solvent A) and 90:10 (vol/vol) isopropanol/acetonitrile was performed as described before (76). As previously outlined, the LC-electrospray ionization (ESI)-HRMS^2^ analyses were achieved by coupling the LC system to a hybrid quadrupole time-of-flight (QTOF) mass spectrometer Agilent 6538 (Agilent Technologies) equipped with dual electrospray ionization (ESI) (37). For quantifications, 2 µL of internal standards (EquiSPLASH LIPIDOMIX, 330731-1EA) was added prior to extraction. The files generated by Agilent (*.d) were converted to the *.mzML format using MSConvert (77). Subsequently, the software MS-DIAL version 5.1 was employed for data processing and lipid annotation (78). The peak height was utilized as the intensity measure for each annotated lipid in the mass spectra. The nomenclature for lipid sub-classes adheres to the definition provided in reference (79). Up to nine assays were carried out for lipid analysis during three independent assays. Outlier analyses were performed via Prism Software V 9.0.

### Laurdan assays

The membrane fluidity of bacteria cultured at 37°C and 18°C was evaluated using Laurdan assays. For these assays, culture samples were diluted to an OD_600nm_ of 1. These cell suspensions (1 mL) were transferred to 2 mL reaction tubes individually and supplemented with 10 µM Laurdan (6-dodecanoyl-2-dimethylaminonaphtalene; Sigma-Aldrich) from a 1 mM Laurdan stock solution dissolved in DMF. Cells were incubated with Laurdan for 20 minutes at the appropriate growth temperatures, avoiding exposure to light. After incubation, the cells were centrifuged for 1 minute at 16,000 × *g* in a× benchtop centrifuge and washed four times with 2 mL of pre-warmed 1× potassium phosphate buffer (PBS). The supernatants were carefully removed by pipetting, and after the final wash, the cells were resuspended in PBS to an OD_600nm_ of 0.5. Then, 150 µL of the stained cells and 1 mM Laurdan were immediately transferred to pre-warmed black flat-bottomed 96-well microtiter plates. Laurdan fluorescence (excitation: 350 nm, emission A: 420–460 nm and emission B: 490–520 nm) was immediately measured using a Clariostar (BMG Labtech) plate reader. The Generalized Polarization(GP) was calculated as follows: GP= (IA-IB)/(IA+IB). Three biological replicates were performed.

### Crystal violet uptake assay

All strains were cultured overnight at both temperatures and then pelleted at 16,000 × *g* for 1 minute. The cells were washed with phosphate-buffered saline (PBS) and resuspended in crystal violet (CV) at 10 µg/mL to an OD_600nm_ of 0.6. After incubation for 10 minutes, the cells were pelleted again, and the supernatant was measured spectrophotometrically at 595 nm. A control sample containing CV without bacteria was used for data normalization. The amount of CV in the supernatant, representing the dye not taken up by the cells, was converted to a percentage as follows [(OD value of CV solution-OD value of sample)/OD value of CV solution] × 100. This procedure was repeated with three biological replicates.

### Statistical analysis

MetaboAnalyst 5.0 (63) was used to estimate variation across the sample group (PLS-DA and volcano plot). For the Volcano plot, the fold change threshold was 3.0 and the *P*-value threshold was 0.05. Significance was analyzed using ANOVA, and Tukey’s was used as a *post hoc* test. Graphs were made using Prism Software V 9.0. The results were considered significant for a *P* value of ≤ 0.05.

## Data Availability

The genomic data are available under BioSample accessions SAMN42933538, SAMN42933539, SAMN42933540, SAMN42933541, and SAMN42933542 on NCBI or via the SRA data PRJNA1142235. All other data associated with this study are provided in the article.

## SUPPLEMENTAL MATERIAL

Fig. S1 spectrum.00757-24-s0001.docx Alignment of 16S-23S rRNA gene spacer region (approximately 600 bp) to confirm the identity of the strains used in this study.

Fig. S2 spectrum.00757-24-s0002.docx Fluidity measurements.

Fig. S3 spectrum.00757-24-s0003.docx Potential desaturase candidates from ABVal2.

Supplemental material spectrum.00757-24-s0004.docx Legends for supplemental movies.

Table S1 spectrum.00757-24-s0005.csv Analyzed alleles to determine ST using both Oxf and Pas schemes.

Video S1 spectrum.00757-24-s0006.avi Twitching Motility at 37°C.

Video S2 spectrum.00757-24-s0007.avi Twitching Motility at 18°C.

ASM does not own the copyrights to Supplemental Material that may be linked to, or accessed through, an article. The authors have granted ASM a non-exclusive, world-wide license to publish the Supplemental Material files. Please contact the corresponding author directly for reuse.
