# Supplementary material for: Lipidomic analyses reveal distinctive variations in homeoviscous adaptation among clinical strains of Acinetobacter baumannii, providing insights from an environmental adaptation perspective
Source: Microbiol Spectr. 2024 Sep 10;12(10):e00757-24. doi: 10.1128/spectrum.00757-24 (PMC11448061; doi:10.1128/spectrum.00757-24)
Supplement: Fig. S1 — Alignment of 16S-23S rRNA gene spacer region (approximately 600 bp) to confirm the identity of the strains used in this study. [file spectrum.00757-24-s0001.docx]

AB5075 1 GTCGTAACAAGGTAGCCGTAGGGGAACCTGCGGCTGGATCACCTCCTTAACGAAAGATTG

ABVal1 1 -----------------------------------------TACCTCCTTACGAAGATTG

ABVal2 1 ---------------------------------------------ACCTTACGACGATTG

ABVal3 1 --------------------------------------------------AGAAAGATTG

ABVal4 1 -----------------------------------------------------ATGATTG

ABVal5 1 ------------------------------------------ACTCCTTACGAAAGATTG

AB5075 61 ACGATTGGTAAGAATCCACAACAAGTTGTTCTTCATAGATGTATCTGAGGGTCTGTAGCT

ABVal1 20 ACGATTGGTAAGAATCCACAACAAGTTGTTCTTCATAGATGTATCTGAGGGTCTGTAGCT

ABVal2 16 ACGATTGGTAAGAATCCACAACAAGTTGTTCTTCATAGATGTATCTGAGGGTCTGTAGCT

ABVal3 11 ACGATTGGTAAGAATCCACAACAAGTTGTTCTTCATAGATGTATCTGAGGGTCTGTAGCT

ABVal4 8 ACGATTGGTAAGAATCCACAACAAGTTGTTCTTCATAGATGTATCTGAGGGTCTGTAGCT

ABVal5 19 ACGATTGGTAAGAATCCACAACAAGTTGTTCTTCATAGATGTATCTGAGGGTCTGTAGCT

AB5075 121 CAGTTGGTTAGAGCACACGCTTGATAAGCGTGGGGTCACAAGTTCAAGTCTTGTCAGACC

ABVal1 80 CAGTTGGTTAGAGCACACGCTTGATAAGCGTGGGGTCACAAGTTCAAGTCTTGTCAGACC

ABVal2 76 CAGTTGGTTAGAGCACACGCTTGATAAGCGTGGGGTCACAAGTTCAAGTCTTGTCAGACC

ABVal3 71 CAGTTGGTTAGAGCACACGCTTGATAAGCGTGGGGTCACAAGTTCAAGTCTTGTCAGACC

ABVal4 68 CAGTTGGTTAGAGCACACGCTTGATAAGCGTGGGGTCACAAGTTCAAGTCTTGTCAGACC

ABVal5 79 CAGTTGGTTAGAGCACACGCTTGATAAGCGTGGGGTCACAAGTTCAAGTCTTGTCAGACC

AB5075 181 CACCATGACTTTGACTGGTTGAAGTTATAGATAAAAGATACATGATTGATGATGTAAGCT

ABVal1 140 CACCATGACTTTGACTGGTTGAAGTTATAGATAAAAGATACATGATTGATGATGTAAGCT

ABVal2 136 CACCATGACTTTGACTGGTTGAAGTTATAGATAAAAGATACATGATTGATGATGTAAGCT

ABVal3 131 CACCATGACTTTGACTGGTTGAAGTTATAGATAAAAGATACATGATTGATGATGTAAGCT

ABVal4 128 CACCATGACTTTGACTGGTTGAAGTTATAGATAAAAGATACATGATTGATGATGTAAGCT

ABVal5 139 CACCATGACTTTGACTGGTTGAAGTTATAGATAAAAGATACATGATTGATGATGTAAGCT

AB5075 241 GGGGACTTAGCTTAGTTGGTAGAGCGCCTGCTTTGCACGCAGGAGGTCAGGAGTTCGACT

ABVal1 200 GGGGACTTAGCTTAGTTGGTAGAGCGCCTGCTTTGCACGCAGGAGGTCAGGAGTTCGACT

ABVal2 196 GGGGACTTAGCTTAGTTGGTAGAGCGCCTGCTTTGCACGCAGGAGGTCAGGAGTTCGACT

ABVal3 191 GGGGACTTAGCTTAGTTGGTAGAGCGCCTGCTTTGCACGCAGGAGGTCAGGAGTTCGACT

ABVal4 188 GGGGACTTAGCTTAGTTGGTAGAGCGCCTGCTTTGCACGCAGGAGGTCAGGAGTTCGACT

ABVal5 199 GGGGACTTAGCTTAGTTGGTAGAGCGCCTGCTTTGCACGCAGGAGGTCAGGAGTTCGACT

AB5075 301 CTCCTAGTCTCCACCAGAACTTAAGATAAGTTCGGATTACAGAAATTAGTAAATAAAGAT

ABVal1 260 CTCCTAGTCTCCACCAGAACTTAAGATAAGTTCGGATTACAGAAATTAGTAAATAAAGAT

ABVal2 256 CTCCTAGTCTCCACCAGAACTTAAGATAAGTTCGGATTACAGAAATTAGTAAATAAAGAT

ABVal3 251 CTCCTAGTCTCCACCAGAACTTAAGATAAGTTCGGATTACAGAAATTAGTAAATAAAGAT

ABVal4 248 CTCCTAGTCTCCACCAGAACTTAAGATAAGTTCGGATTACAGAAATTAGTAAATAAAGAT

ABVal5 259 CTCCTAGTCTCCACCAGAACTTAAGATAAGTTCGGATTACAGAAATTAGTAAATAAAGAT

AB5075 361 TGAGATCTTGGTTTATTAACTTCTGTGATTTCATTATCACGGTAATTAGTGTGATCTGAC

ABVal1 320 TAAGATCTTGGTTTATTAACTTCTGTGATTTCATTATCACGGTAATTAGTGTGATCTGAC

ABVal2 316 TGAGATCTTGGTTTATTAACTTCTGTGATTTCATTATCACGGTAATTAGTGTGATCTGAC

ABVal3 311 TAAGATCTTGGTTTATTAACTTCTGTGATTTCATTATCACGGTAATTAGTGTGATCTGAC

ABVal4 308 TGAGATCTTGGTTTATTAACTTCTGTGATTTCATTATCACGGTAATTAGTGTGATCTGAC

ABVal5 319 TGAGATCTTGGTTTATTAACTTCTGTGATTTCATTATCACGGTAATTAGTGTGATCTGAC

AB5075 421 GAAGACACATTAACTCATTAACAGATTGGCAAAATTGAGTCTGAAATAAATTGTTCACTC

ABVal1 380 GAAGACACATTAACTCATTAACAGATTGGCAAAATTGAGTCTGAAATAAATTGTTCACTC

ABVal2 376 GAAGACACATTAACTCATTAACAGATTGGCAAAATTGAGTCTGAAATAAATTGTTCACTC

ABVal3 371 GAAGACACATTAACTCATTAACAGATTGGCAAAATTGAGTCTGAAATAAATTGTTCACTC

ABVal4 368 GAAGACACATTAACTCATTAACAGATTGGCAAAATTGAGTCTGAAATAAATTGTTCACTC

ABVal5 379 GAAGACACATTAACTCATTAACAGATTGGCAAAATTGAGTCTGAAATAAATTGTTCACTC

AB5075 481 AAGAGTTTAGGTTAAGCAATTAATCTAGATGAATTGAGAACTAGCAAATTAACTGAATCA

ABVal1 440 AAGAGTTTAGGTTAAGCAATTAATCTAGATGAATTGAGAACTAGCAAATTAACTGAATCA

ABVal2 436 AAGAGTTTAGGTTAAGCAATTAATCTAGATGAATTGAGAACTAGCAAATTAACTGAATCA

ABVal3 431 AAGAGTTTAGGTTAAGCAATTAATCTAGATGAATTGAGAACTAGCAAATTAACTGAATCA

ABVal4 428 AAGAGTTTAGGTTAAGCAATTAATCTAGATGAATTGAGAACTAGCAAATTAACTGAATCA

ABVal5 439 AAGAGTTTAGGTTAAGCAATTAATCTAGATGAATTGAGAACTAGCAAATTAACTGAATCA

AB5075 541 AGCGTTTTGGTATGTGAATTTAGATTGAAGCTGTACGGTGCTTAAGTGCACAGTGCTCTA

ABVal1 500 AGCGTTTTGGTATGTGAATTTAGATTGAAGCTGTACGGTGCTTAAGTGCACAGTGCTCTA

ABVal2 496 AGCGTTTTGGTATGTGAATTTAGATTGAAGCTGTACGGTGCTTAAGTGCACAGTGCTCTA

ABVal3 491 AGCGTTTTGGTATGTGAATTTAGATTGAAGCTGTACGGTGCTTAAGTGCACAGTGCTCTA

ABVal4 488 AGCGTTTTGGTATGTGAATTTAGATTGAAGCTGTACGGTGCTTAAGTGCACAGTGCTCTA

ABVal5 499 AGCGTTTTGGTATGTGAATTTAGATTGAAGCTGTACGGTGCTTAAGTGCACAGTGCTCTA

AB5075 601 AACTGAAATGTTGAAGTTACTAACTTGTAGGTAACATCGACTGTTTGGGGTTGTATAGTC

ABVal1 560 AACTGAAATGTTGAAGTTACTAACTTGTAGGTAACATCGACTGTTTGGGGTTGTATAGTC

ABVal2 556 AACTGAAATGTTGAAGTTACTAACTTGTAGGTAACATCGACTGTTTGGGGTTGTATAGTC

ABVal3 551 AACTGAAATGTTGAAGTTACTAACTTGTAGGTAACATCGACTGTTTGGGGTTGTATAGTC

ABVal4 548 AACTGAAATGTTGAAGTTACTAACTTGTAGGTAACATCGACTGTTTGGGGTTGTATAGTC

ABVal5 559 AACTGAAATGTTGAAGTTACTAACTTGTAGGTAACATCGACTGTTTGGGGTTGTATAGTC

AB5075 661 AAGTAATTAAGTGCATGTGGTGGATGCCTTGGCAGTCAGAGGCGATGAAAGACGTGATAG

ABVal1 620 AAGTAATTAAGTGCATGTGGTGGATGCCTTGGCAGTCAGAGGCGATGAAAGACGTGATAG

ABVal2 616 AAGTAATTAAGTGCATGTGGTGGATGCCTTGGCAGTCAGAGGCGATGAAAGACGTGATAG

ABVal3 611 AAGTAATTAAGTGCATGTGGTGGATGCCTTGGCAGTCAGAGGCGATGAAAGACGTGATAG

ABVal4 608 AAGTAATTAAGTGCATGTGGTGGATGCCTTGGCAGTCAGAGGCGATGAAAGACGTGATAG

ABVal5 619 AAGTAATTAAGTGCATGTGGTGGATGCCTTGGCAGTCAGAGGCGATGAAAGACGTGATAG

AB5075 721 CCTGCGAAAAGCTCCGGGGAGGCGGCAAATATCCTTTGATCCGGAGATGTCTGAATGGGG

ABVal1 680 CCTGCGAAAAGCTCCGGGGAGGCGGCAAATATCCTTTGATCCGGAGATGTCTGAAC----

ABVal2 676 CCTGCGAAAAGCTCCGGGGAGGCGGCAAATATCCTTTGATCCGGAGATGTCTGATG----

ABVal3 671 CCTGCGAAAAGCTCCGGGGAGGCGGCAAATATCCTTTGATCCGGAGATGTCTGATGGGG-

ABVal4 668 CCTGCGAAAAGCTCCGGGGAGGCGGCAAATATCCTTTGATCCGGAGATGTCTGATGG---

ABVal5 679 CCTGCGAAAAGCTCCGGGGAGGCGGCAAATATCCTTTGATCCGGAGATGTCTGATGGGGG

AB5075 781 GAACCC-

ABVal1 735 -------

ABVal2 731 -------

ABVal3 729 -------

ABVal4 724 -------

ABVal5 739 GAACCCC

**Supplementary figure 1**. Alignement of 16S-23S rRNA gene spacer region (approximately 600 bp) to confirm the identity of the strains used in this study.
