# Supplementary material for: Lipidomic analyses reveal distinctive variations in homeoviscous adaptation among clinical strains of Acinetobacter baumannii, providing insights from an environmental adaptation perspective
Source: Microbiol Spectr. 2024 Sep 10;12(10):e00757-24. doi: 10.1128/spectrum.00757-24 (PMC11448061; doi:10.1128/spectrum.00757-24)
Supplement: Fig. S2 — Fluidity measurements. [file spectrum.00757-24-s0002.docx]

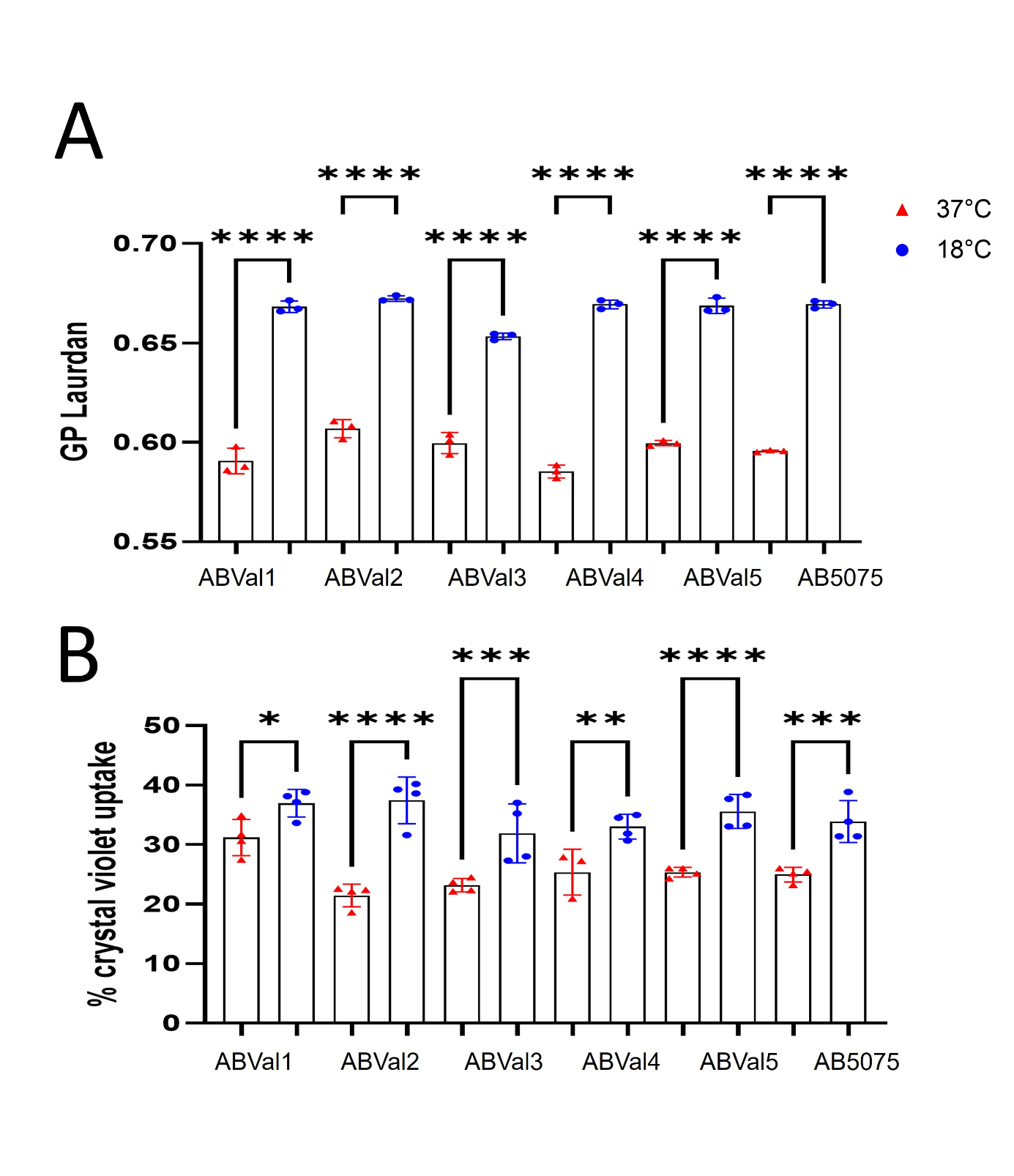


**Supplementary figure 2.** Measures of the membrane fluidity. (A), generalized polarization (GP) measurements at 37°C and 18°C. The results correspond to n = 3 biologically independent samples. (B), percentage of crystal violet uptake at 37°C and 18°C. The results correspond to n = 4 biologically independent samples. Statistical significances were determined by a two-tailed student’s t test (****, p ≤ 0.0001; ***, p ≤ 0.001; **, p ≤0.01; *, p ≤0.05).
