# Supplementary material for: Lipidomic analyses reveal distinctive variations in homeoviscous adaptation among clinical strains of Acinetobacter baumannii, providing insights from an environmental adaptation perspective
Source: Microbiol Spectr. 2024 Sep 10;12(10):e00757-24. doi: 10.1128/spectrum.00757-24 (PMC11448061; doi:10.1128/spectrum.00757-24)
Supplement: Fig. S3 — Potential desaturase candidates from ABVal2. [file spectrum.00757-24-s0003.docx]

DesA2
MNAQVSVTDLFSREEIQELTEPSDAYGAWAVASTWAVIGGTFASLIMMWDYLPSWGKLLACMLALAVLAGRQLCLAILMHDASHKSLFKNKKINDFVGEWLCARPIWNDLQKYRVHHVRHHAKTSTPDDPDLSLVAGFPVSKQSLTRKFLRDLTGITGLKFSLGRVLMDLDVMKWTVANDQIWLDRSDKNFVDYAKSIAKNSTGAIATNLLLYGVLKACGQQRFYWLWPLAYLTPFPLFLRIRSMAEHAGMQTSNTALTNTRTTRAGWIARSFVAPIHVNYHMEHHLMASVPYFKLPRMHKILRERGHVPTPPSYFEVIHILSSKQELTN*


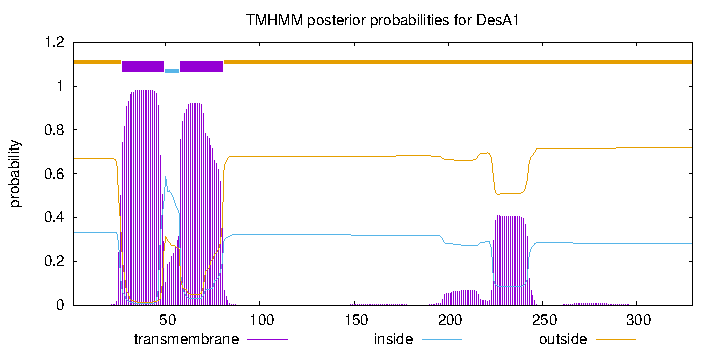


DesA3
MTYIYKNPAGMTDSEKTEHIKKVVTAEGVALRKRHPILNHQNAIGAMILFISLVGMIATAVLYINHQLSAWFAIPIIAFFASLTHELEHDLIHWMYFRKKPWAHHLMMGLVWLARPSTINPWKRRELHFNHHKNSGTEVDLEERALTNGEQWSIRRLIAIGDNGLAVLFRIISASNWTVRKVIFKRAFMAYFPLGIIHWSLWYIFLGFHAVDAVLSWANAPIAWSATTLNIMHVVNILTVVWVAPNVLRTFCLHFVTSNMHYYGDVELGNVIQQTQVLKPWWMMPFQLFCFNFGSTHAIHHFVVKEPFYIRQMTAPVAHKVMRDMGVRFNDVGTFKRANRWNINDLSESKS*


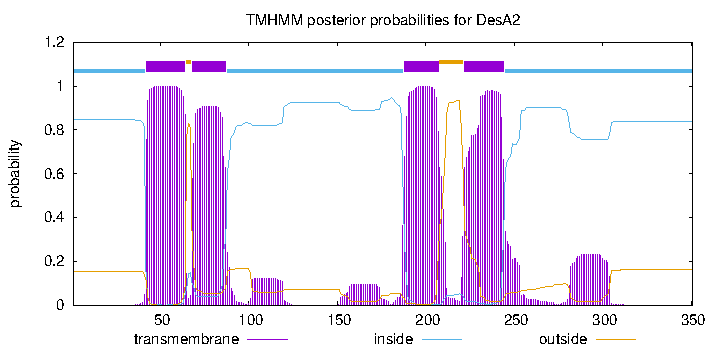


DesB2
MNMALKVSMASKSAHLTPEQIEEFGRRVEQIRQDVMQSLGEQDAKYIYKVRNFVRYTEIASRGMLMFGGWIPPVWLLGTGLLGISKIVENMELGHNVMHGQFDWLNEPSLNGNTYDWDTIASGDDWRETHNYVHHTYTNIVGKDHDVGYGILRVSDQQKWEPRHLFNIPLALQLMFFFEWYVGVQNLHLEDALVYKTKSWKQVWKDAAKVRKKATRQILKDYVFFPVISGPMFLPVFAGNVVANIIRNLWSSAVIFNGHFTEDAETFEPDNTDTETKAEWYLRQIRGSSNFSGTEWLHFMSGNLSHQIEHHLFPDMPANRYKEVAPKIKALCAEYGINYNEANFMRQFWSVWVRLAKCSLPNHTTAKVMQTLEKLKAKFKFA*


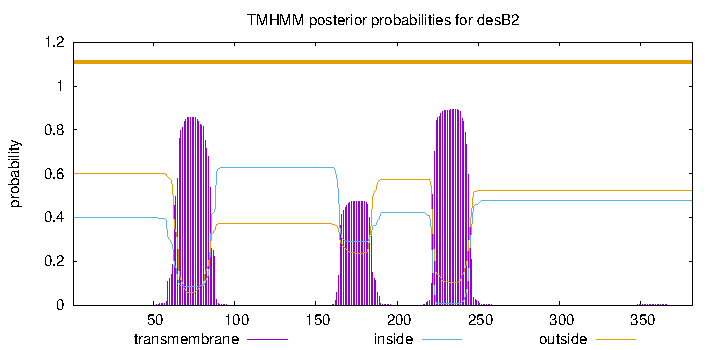


DesA :
MTRFLGALPDMTSAPLKAPINWTASITLIGLPILAAIIIPIYAYYYDFSVSAWVSLFFLLALSSMGITAGYHRLWAHRAYEASLPLKILLMIGGTFAVQNSILFWASGHRTHHRHVDDVDEDPYSIERGFWYAHMGWMIRDHSPSEPDFKNAPDLLNDKLVMFQHKYYALMVVAVHVGILGLVGWATGDLWGVVLLGGLLRLIISHQVTFFINSLCHMFGKRPYTDENSARDNFWLAIATWGEGYHNYHHIFQYDYRNGVKWWQYDPTKWLIWTCSKIGLAKNLRRIPSFNIKKAELAMKFKYAEQDLAIYGHDVNADISQMKQRIAQEYEAFTHTLNDWAKLKEQELQA
KKAAMAEKIHKMDHKLKVDFQLLEHRLSHHRECLETLMRNIKKNTNVVPD


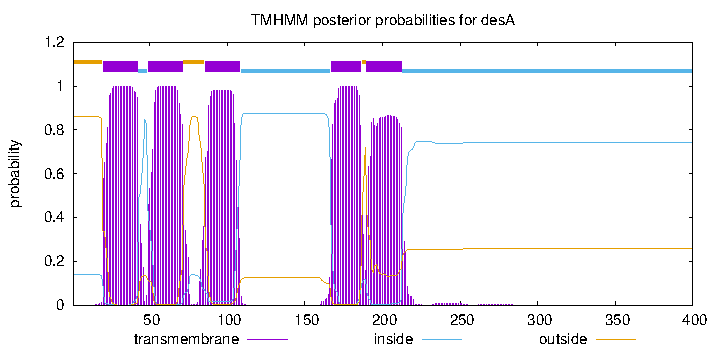


DesB
MNMPVKVEYFKNPKNRDLTPAELEAFAKELDQIKQEVLDDLGEKDAKYIRRVYAAIRYSSFLGRACLFAGWFPPAWILGTGLLGFSKIMENMELGHNVMHGQYDWMNDPKFNGQTYEWDTVGTSDNWRQTHNYKHHTYTNIKGIDDDIGYGLLRLFPEQRWKPGFLLQPIYSIPFCLLFQWGVAIQNLEIGRVLYKRKTKAQFLEELKPVNKKIGKQLFKDYVFFPLIAGPAALPVFTGNLVANGLRNIWTFSIIFCGHFTKDAEVFPKSVLQEESRGHWYMRQIRGSSNLTGSEAFHILSGHLSHQIEHHLFPDIPARRYRQMAPKVEAVCKKYGLNYNNASFVKQFGQ
VVGRIVKYAFPFKK*


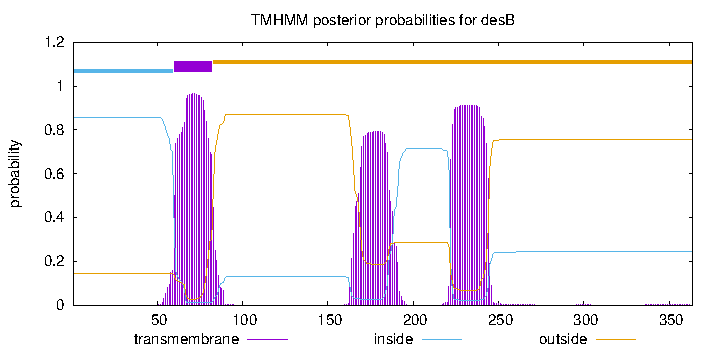


**Supplementary figure 3.** Potential desaturase candidates from ABVal2 include DesA and DesB, which have been previously characterized (1). DesA2, DesA3, and DesB2 are uncharacterized. The three conserved histidine-rich motifs (“HX3 (or X4)H,” “HX2 (or X3)HH,” and “H/QX2 (or X3)HH”) are highlighted in red. Below each sequence are the predicted transmembrane regions for that sequence, as determined by the TMHMM service (2).

1. Adams FG, Pokhrel A, Brazel EB, et al (2021) *Acinetobacter baumannii* fatty acid desaturases facilitate survival in distinct environments. ACS Infect Dis acsinfecdis.1c00192

2. Krogh A, Larsson B, Heijne G von, et al (2001) Predicting transmembrane protein topology with a hidden Markov model: application to complete genomes. J Mol Biol 305:567–80
