# Supplementary material for: Lipidomic analyses reveal distinctive variations in homeoviscous adaptation among clinical strains of Acinetobacter baumannii, providing insights from an environmental adaptation perspective
Source: Microbiol Spectr. 2024 Sep 10;12(10):e00757-24. doi: 10.1128/spectrum.00757-24 (PMC11448061; doi:10.1128/spectrum.00757-24)
Supplement: Supplemental material — Legends for supplemental movies. [file spectrum.00757-24-s0004.docx]

**Supplemental movie, Video 1.** Twitching Motility at 37°C. The video showcases twitching motility observed under a magnification of X100, captured every 2 seconds over a duration of 2 minutes, following 4 hours of incubation at 37°C.

**Supplemental movie, Video 2.** Twitching Motility at 18°C. The video showcases twitching motility observed under a magnification of X100, captured every 2 seconds over a duration of 2 minutes, following 4 hours of incubation at 18°C.
